# Resistance mutations in protease gene at baseline are not related to virological failure in patients treated with darunavir/ritonavir monotherapy

**DOI:** 10.7448/IAS.17.4.19760

**Published:** 2014-11-02

**Authors:** Angela Gutierrez-Liarte, Ana Gomez-Berrocal, Carmen Saez, Jorge Valencia, Ignacio Santos, Jesus Sanz

**Affiliations:** Infectious Diseases, Hospital de La Princesa, Madrid, Spain

## Abstract

**Introduction:**

Monotherapy with darunavir plus ritonavir (DRV/r) is a good maintenance strategy for suppressed HIV-infected patients. The clinical trials designed to prove the efficacy of PI/r do not include patients with resistance mutation in protease gene [1,2]. Sometimes in routine practice, basically to avoid NRTIs toxicity, monotherapy with DRV/r is used despite PI resistance mutations. The aim of this study is to know the effect of previous protease resistance mutation on DRV/r monotherapy efficacy.

**Materials and Methods:**

We designed an observational cohort study of adults in treatment with DRV/r monotherapy in a tertiary Spanish hospital since 2011 to 2014. Demographic data and clinical outcomes were described. The analysis of efficacy was done according to the snapshot algorithm (defining virological failure as viral load >50 copies/mL, ITTe, at 48 and 96 weeks). We analyzed the difference of efficacy between patients with and without baseline resistance mutations at 48 and 96 weeks by using the χ^2^ test; and during the follow-up by using the Kaplan–Meier test. The statistical analysis was done with SPSS 17.0.

**Results:**

Eighty-nine patients were included in the cohort but 14 were excluded because they had not reached more than six months with monotherapy. The cohort was composed mainly by men (78%), the medium age was 51 years (SD±10), 35% were MSM and 19% were former IDU. Twenty-four patients (35%) had a previous diagnosis of AIDS. The mean time taking NRTIs was 10.5 years (SD±5.4). Sixty-four patients (85%) had been treated with PI in the past. Previous failure with PI had been reported in 15 (20%). A resistance mutation test had been done at baseline in 45 patients (51%). Twenty-two patients (29%) had some mutations in protease gene, 10 patients (13%) had major mutations and 1 patient had some mutations of resistance for darunavir (I64V). At 48 weeks, 93% (CI 95% 86–98%) had VL<50 copies/mL, and 79% (CI 95% 67–89%) at 96 weeks. There were not differences between patients with or without resistance mutations (p=0.53). After a median follow-up of 70 weeks, 88% of patients remain free of virological failure and there were not differences between both groups.

**Conclusions:**

According to these data, previous resistance mutations in the protease gene, which do not affect darunavir, are not related with the efficacy in patients treated with DRV/r monotherapy.

## References

[1] Arribas JR, Horban A, Gerstoft J, Fätkenheuer G, Nelson M, Clumeck N (2010). The MONET trial: darunavir/ritonavir with or without nucleoside analogues, for patients with HIV RNA below 50 copies/ml. AIDS.

[2] Valantin MA, Lambert-Niclot S, Flandre P, Morand-Joubert L, Cabié A, Meynard JL (2012). Long-term efficacy of darunavir/ritonavir monotherapy in patients with HIV-1 viral suppression: week 96 results from the MONOI ANRS 136 study. J Antimicrob Chemother.

